# Evaluating Frequency, Diagnostic Quality, and Cost of Lyme Borreliosis Testing in Germany: A Retrospective Model Analysis

**DOI:** 10.1155/2012/595427

**Published:** 2011-12-27

**Authors:** I. Müller, M. H. Freitag, G. Poggensee, E. Scharnetzky, E. Straube, Ch. Schoerner, H. Hlobil, H.-J. Hagedorn, G. Stanek, A. Schubert-Unkmeir, D. E. Norris, J. Gensichen, K.-P. Hunfeld

**Affiliations:** ^1^Bacteriologic Infection Serology Study Group of Germany (BISSGG), Central Laboratory, Institute for Laboratory Medicine, Northwest Medical Centre, Academic Teaching Hospital, Medical Faculty, Johann Wolfgang Goethe-University, Steinbacher Hohl 2-26, 60488 Frankfurt am Main, Germany; ^2^Department of General Practice and Family Medicine, Jena University Hospital, Bachstraße 18, 07743 Jena, Germany; ^3^Surveillance Unit, Department of Infectious Disease Epidemiology, Robert Koch-Institute, DGZ-Ring 1, 13086 Berlin, Germany; ^4^Deutsche Angestellten-Krankenkasse (DAK), Nagelsweg 27-31, 20097 Hamburg, Germany; ^5^Department of Molecular Microbiology and Immunology, Johns Hopkins Bloomberg School of Public Health, Baltimore, North Wolfe Street, MD 21205, USA

## Abstract

*Background*. Data on the economic impact of Lyme borreliosis (LB) on European health care systems is scarce. This project focused on the epidemiology and costs for laboratory testing in LB patients in Germany. *Materials and Methods*. We performed a sentinel analysis of epidemiological and medicoeconomic data for 2007 and 2008. Data was provided by a German statutory health insurance (DAK) company covering approx. 6.04 million members. In addition, the quality of diagnostic testing for LB in Germany was studied. *Results*. In 2007 and 2008, the incident diagnosis LB was coded on average for 15,742 out of 6.04 million insured members (0.26%). 20,986 EIAs and 12,558 immunoblots were ordered annually for these patients. For all insured members in the outpatient sector, a total of 174,820 EIAs and 52,280 immunoblots were reimbursed annually to health care providers (cost: 2,600,850€). For Germany, the overall expected cost is estimated at 51,215,105€. However, proficiency testing data questioned test quality and standardization of diagnostic assays used. *Conclusion*. Findings from this study suggest ongoing issues related to care for LB and may help to improve future LB disease management.

## 1. Introduction

Lyme borreliosis (LB) is a vector-borne disease that is transmitted by ixodid ticks and is caused by the spirochetes of the *Borrelia (B.) burgdorferi* sensu lato (s.l.) complex. The 5 genospecies that are currently considered to be human pathogens are *B. burgdorferi* sensu stricto (s.s.), *B. afzelii*, *B. garinii*, *B. spielmanii*, and the proposed but not yet validated novel species *B. bavariensis* [1]. Over the last 20 years, LB has been recognized as a major public health problem in the United States and in Europe [2, 3]. Part of this status is related to variation in symptoms and the clinical picture of the disease [4]. In addition, further insecurity exists with the differential diagnostic considerations in LB patients, the natural trajectory of treated and untreated LB, and interpretation of diagnostic test results [2, 5, 6]. In Europe, the incidence of Lyme borreliosis is estimated to range from 0.6 to 155/100,000 [4, 7]. In Germany, the estimation of 60,000–100,000 incident cases per year is based on an older seroprevalence study in a single region [8]. Mandatory reporting of new LB cases was established in 2002, but only in the six new federal states of the northeastern part of Germany. About 5,221 new cases of LB are reported for these states per year [9]. Little is known about the true incidence and distribution of LB in the other parts of Germany.

LB can manifest itself progressively as a multisystem disorder exhibiting a broad spectrum of clinical symptoms [4, 10]. The disease is usually diagnosed clinically based on a characteristic clinical picture, a history of tick bite, and the diagnosis then can be supported further by serological testing. However, both false negative and false positive serologic test results do occur, and together with a lack of standardization of current diagnostic methods can clearly impede a clear and concise diagnosis [11]. Moreover, current law in most European countries does not require profound clinical evaluation of such commercially available diagnostic test kits for LB prior to market registration. Most significant, however, is the high seroprevalence of anti-*B. burgdorferi* antibodies that correlates with manifest disease in only a minority of patients. Therefore, serology should only be used to confirm but not to primarily establish the diagnosis of LB. In addition, the antibody titers on followups do not reflect the success of antibiotic treatment [12]. These factors can lead to misdiagnosis and mismanagement of LB and patients after tick bites and/or unspecific symptoms. As reported in the US, these events may lead to inappropriate care for patients including adverse effects and unnecessary financial cost [2, 5].

The situation in Germany is less obvious. Currently, little data is available on health care utilization in Germany for patients with confirmed or suspected LB such as performance of diagnostic and therapeutic measures including those for unspecific chronic conditions which are attributed to LB by patients or their physicians. The aim of the German investigation on Lyme borreliosis: evaluation of therapeutic and diagnostic cost (GILEAD) study is a step-wise analytic approach to estimate the amount of diagnostic testing, assay quality, and cost related to manifest and suspected LB in Germany. In this analysis, we explore the relative frequency of diagnostic testing, the number of incident and prevalent diagnoses, and the cost of laboratory diagnostics by analyzing German health insurance claims' data. In addition, we performed a meta-analysis of results obtained during the regular German LB serology proficiency testing program to learn more about the accuracy and reliability of currently used serological tests in Germany. This was done to evaluate the hypothesis that, although guidelines for the diagnostic management of LB with well-defined diagnostic algorithms for suspected LB cases have been established in Germany [13–15], relatively high volumes of diagnostic efforts (i.e., serologic testing) related to LB are being performed repeatedly without necessarily adding any benefit to the management of such patients. 

## 2. Materials and Methods

### 2.1. Analysis of Health Insurance Datasets

The basic dataset consists of health insurance data from a German statutory health insurance company (Deutsche Angestellten-Krankenkasse, DAK) which covers approx. 6.04 million individuals all over Germany. The population insured by DAK consists of more women than men (ratio 1.8 : 1). In a first step, relevant international classification of diseases (ICD 10-GM, 2004) diagnoses for Lyme borreliosis were defined as follows: ICD A69.2 for Lyme-specific erythema chronicum migrans, GO1* for LB-related meningitis, G63.0 for LB-related polyneuropathy, and M01.2 for LB-related Arthritis. Claims' data of the years 2007 and 2008 were derived from the underlying datasets (patient data, ambulatory treatment data, and medication data). In accordance with legal data protection requirements, all personal data were exclusively handled by DAK. Analyses were based on subject-specific data which did not allow the identification of individual persons. Informed consent is not required for these analyses in Germany. The quality of the data was checked for completeness, correct usage of inclusion criteria, and plausibility prior to analysis according to existing standards [16, 17]. Individuals insured at least since January 1, 2006, or January 1, 2007, respectively, in whom at least one laboratory diagnostic procedure performed for LB in either year 2007 or 2008, were included in our analyses. The diagnostic procedures according to the general laboratory health insurance claim code (“Einheitlicher Bewertungsmassstab”, EBM [18]) included laboratory claim numbers 32586 (*B. burgdorferi* antibody/enzyme-linked immune assay, ELISA), 32662 (*B. burgdorferi* antibody/western blot), and/or 32743 (culture of *B. burgdorferi*). Individuals already having a coded diagnosis of Lyme borreliosis in 2006 were excluded from the analysis. We also extracted patient data (subject specifier, gender, year of birth, code for current residence, date of begin and end of insurance), ambulatory treatment data (subject specifier, quarter of the year, start and end of treatment period, diagnoses and diagnoses' assurance level, EBM codes, EBM date, area of specialty of physician), and medication data (subject specifier, date of prescription, agent, amount prescribed, cost).

### 2.2. Collecting Data from the German Lyme Disease Proficiency Testing Program

From March 2006 to November 2008, six LB serology proficiency testing surveys were conducted in Germany by the central reference laboratory for bacteriologic serodiagnostics of the Bacteriologic Infection Serology Study Group of Germany (BISSGG) now situated at the Institute of Laboratory Medicine, Northwest Medical Centre, Frankfurt/Main, in cooperation with the WHO Collaborating Centre for Quality Assurance and Standardization in Laboratory Medicine e.V. (INSTAND e.V.), Düsseldorf, and with the 9 reference laboratories of the BISSGG. The organisation and structure of the German proficiency testing program for bacteriologic infection serology is summarised elsewhere in more detail [11, 19, 20]. 

### 2.3. Selection of Serum Samples

Twelve serum samples were obtained from voluntary donors according to previously published strict criteria and after obtaining written informed consent [11, 19]. All subjects were clinically evaluated by experienced physicians. Six serum samples contained specific antibodies against *B. burgdorferi* as determined by various commercial test systems. All antibody-positive donors could recall a known history of a recent tick bite or active or past LB, which also had been documented in the medical records of these patients by their physicians. Six samples tested negative for specific antibodies against *B. burgdorferi *and were used as negative controls. Current or very recent LB was excluded in these donors by careful physical examination, evaluation of patients' medical histories, and review of the medical records provided by the referring physicians. Two of the six negative samples contained anti-*T. pallidum* antibodies and were obtained from a donor with a past syphilis infection and a donor eight weeks after appropriate therapy.  Table 1 provides a detailed description of the clinical data available for all twelve samples.

### 2.4. Study Conditions and Evaluation of Proficiency Testing Results

**Figure 1:**
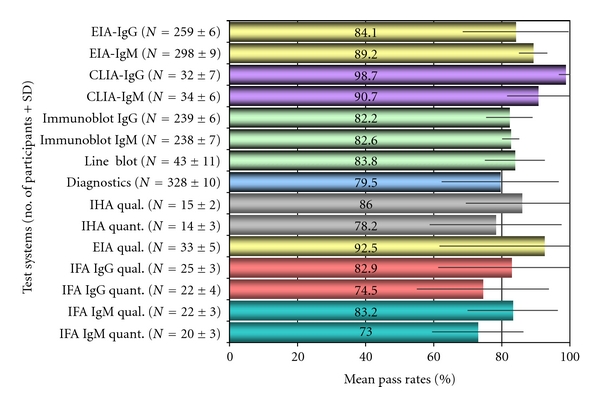
Average number of participants and mean pass rates (%) with standard deviations (bars) for different assay systems as observed between 2006 and 2008 in the German LB proficiency testing program.

**Figure 2:**
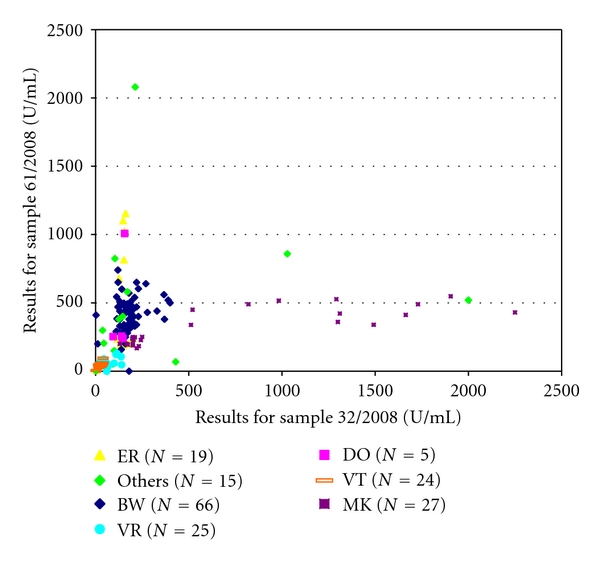
Youden‘s plot of different quantitative IgG-EIA results (U/mL) as obtained in two samples (no. 32 and 61) used for LB proficiency testing in 2008. For clinical information of samples, see Table 1. (ER, BW, DO, MK, VR, VT, others: anonymized abbreviations of different commercial EIA manufacturers).

Assessment of reference test results was performed according to the current guidelines of the German Medical Association and the standard operating procedures of INSTAND for proficiency testing in infection serology as recently described in more detail [11, 19]. Qualitative and quantitative reference test results (Table 1) were determined for each pair of serum samples by 3 to 9 different specialised laboratories or university laboratories of the BISSGG with extensive expertise in the field of serodiagnostic testing for LB. Participation in the LB serology proficiency testing programs was not mandatory, but participating laboratories were required to register at INSTAND prior to their involvement. No preexisting criteria were established to exclude any laboratories from the survey. All participants were instructed to treat samples as routine samples and to perform their established serological test methods on the distributed samples blinded to additional clinical information to guarantee maximum objectivity. Qualitative and quantitative results had to be reported together with the methods used, the lot number and test manufacturer, and the laboratory machinery utilized [19]. Moreover, the laboratories reported interpretative statements as to whether the test constellation suggested a possible *Borrelia* infection and whether an early or late phase of infection was suspected. Reports were made in standardised form on defined evaluation sheets by use of a predefined code to permit statistical analysis after the surveys [19]. Only one test result per test method (indirect immune fluorescence assay: IFA, indirect hemagglutination assay: IHA, enzyme Immunoassay: EIA, chemoluminescence assay: CLIA, line blot, immunoblot, etc.) was reported to INSTAND by each participant. Participants were requested to return their reports to INSTAND for further computer-assisted evaluation of results within 10 days after receipt of samples [11, 19]. Qualitative results from participants were accepted as being *accurate* if their reported test results were congruent with the modal as determined by the reference laboratories (Table 1; Figure 1). Because the quantitative EIA results reported were so heterogeneous (Figure 2) owing to the different quantification methods of the test manufacturers, these results were not included in the evaluation listed below. Quantitative results of classical titre tests (IHA, IFA) were accepted as being *accurate *provided that results from participants were reported within a range of ±2 log_2_ unit dilutions around the median of the test results obtained by the reference laboratories. A qualifying certificate was forwarded to successfully participating laboratories for each parameter under the condition that their microbiological commentary, and qualitative and quantitative test results, for both samples using established assay systems met the above-listed criteria [11, 19].

### 2.5. Statistical Analysis

All included claims' data were analyzed both within the entire group of individuals and within the group of patients with a coded incident diagnosis of A69.2 in the year 2007 or 2008. To avoid misrepresentation, the population was analysed by age and gender and standardized with the general population of Germany (according to “Empfehlungen der Ersatzkassen und ihrer Verbände zur Umsetzung des §20 SGB V”). The numbers of all insured individuals were provided by gender, 5-year age groups, and 5-digit residence codes and used to compare to the group with a coded diagnosis of LB and/or a borreliosis test. Data analyses were descriptive and stratified by sex and standardized by age. Counts and incidence rates for LB-related diagnostic testing were calculated. All data including proficiency testing results were reported as absolute numbers, means, modals, and percentages including standard deviations (SDs) as indicated and helpful (Figure 1). In addition, mean pass rates (Table 2; Figure 1) were calculated from the specific pass rates of the individual surveys performed biannually.

## 3. Results

### 3.1. Results from the German Proficiency Testing Program

From March 2006 to October 2008, between 360 and 392 microbiological laboratories (mean: *N* = 381, SD = 11), including hospital laboratories, independent laboratories, physicians' office laboratories, and manufacturers of commercially available diagnostic LB assays took part in each of the six surveys that were held. Tests employed were those used routinely for the serodiagnosis of LB in the participating laboratories. Figure 1 gives an overview on the frequency of the various test systems used by the participants during the surveys. The laboratories most frequently performed a two-tier protocol, beginning with a sensitive EIA or CLIA screening (mean: *N* = 312 (81.9%), SD = 6.9) followed by immunoblot or line blot confirmation of the results (mean: *N* = 282 (74.0%), SD = 9), in compliance with the current recommendations of the CDC and most European scientific expert opinions [13, 21, 22]. On average, for direct immunoglobulin class-specific analysis of samples, IgG- and/or IgM-EIA were used by 259 (SD = 6) and 298 (SD = 9) of the participants, respectively, during the six surveys. An immunoblot confirmatory assay for IgG- and/or IgM-antibodies was performed by 239 (SD = 6) and 238 (SD: *N* = 7) of the laboratories, respectively. Some other traditional or more recently introduced serological test methods were employed much more rarely: IHA, mean: *N* = 15 (3.9%), IFA, mean: *N* = 23.5 (6.2%), polyvalent EIA, mean: *N* = 33 (8.7%), CLIA, mean: *N* = 33 (8.7%). Interestingly, there was a steady increase for new recombinant tests or hybrid assays (using native and/or recombinant protein preparations) and new analytical test formats such as CLIA and line blots, from 5.8% and 8.5% in 2006 to 10.5% and 16.4% in 2008 (data not shown). 

### 3.2. Accuracy of Test Results

Characteristics of the selected serum samples applied in the German LD proficiency testing program as determined by the nine reference laboratories are depicted in Table 1. The percentages of laboratories that reported correct results with their routinely applied assay systems on the twelve serum samples sent out for testing in the six surveys of the German LD proficiency testing program from 2006–2008 are summarised in Table 2 and Figure 1, as individual pass rates per method and sample or as mean pass rates over time. IgG tests (mean pass rate: 92.1%, range 41.7 to 100%) were only slightly more accurate than those for IgM tests (mean pass rate: 90.3%, range: 11.5 to 100%). Mean pass rates for immunoblot testing (mean pass rates: 92.6%, range: 75 to 100%) were less accurate than those for EIA-testing (EIA mean pass rates: 92.5%, range 53.5–100%). Less frequently used tests such as polyvalent EIA, IFA, and IHA demonstrated mean pass rates for qualitative test results in the range of 11.8 to 99.6% (mean: 86.6%), 41.7 to 100% (mean pass rates: 86.5%), and 66.7 to 100% (mean pass rate: 90.6%). Newly introduced test systems such as CLIA and line blot showed a variable performance with pass rates from 11.5 to 100% (mean pass rate: 89.0%).

Serum samples from patients with long-lasting immune responses (Lyme arthritis, lymphocytoma, recent recurrent tick bites) showed that predominant IgG responses (32/2006, 62/2007, 61/2008) were reproducibly detected and correctly interpreted by most of participants. In contrast to this finding, samples from patients with shorter disease duration or lower titers of specific IgM and IgG antibodies, such as sera from neuroborreliosis cases (62/2006 and 32/2008), past infections (serum scar: sample 21/2007), and cross-reacting samples such as sera from syphilis patients (61/2007), posed more significant diagnostic problems. Here, especially IHA and IFA tended to fail in identifying these samples correctly (Tables 1 and 2).

Our observations with classical titer tests revealed that although the calculated median IFA and IHA titers of the reference laboratories and those of the participants in the majority of cases generally varied only for one to two log_2_ unit dilutions around the median; the ranges of titers in the group of participants revealed an enormous variability of test results (data not shown). Similarly, the quantitative results in EIAs demonstrated a very low level of interassay standardisation in all trials, resulting in a rather high heterogeneity of reported quantitative results (Figure 2). This finding is largely due to methodological differences of commercially manufactured assays and the variable methods of quantification (values of optical density (OD), indices, titers, U/mL) used. Similar to previous investigations [11], we decided not to include quantitative EIA results in the final evaluation of the proficiency testing surveys because of the obvious lack of assay standardisation.

**Figure 3:**
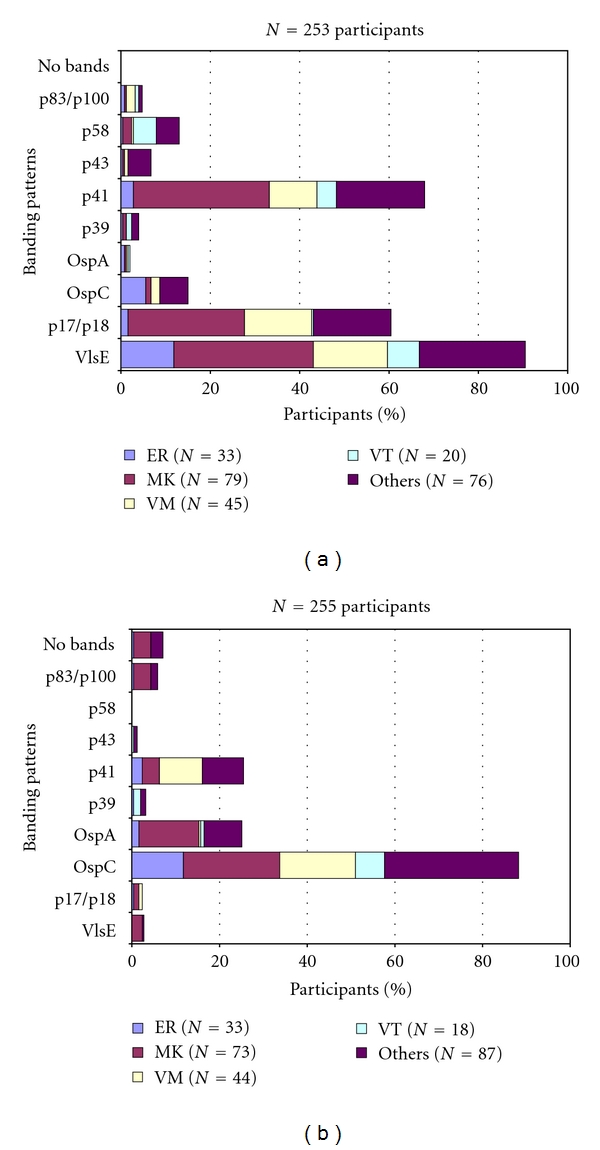
Recovery of LB-specific IgM- (a) and IgG- (b) immunoblot banding patterns (reported borrelial antigen bands: p83/100, p58, p43, p41, p39, OspA, OspC, p17/18, VlsE) as obtained from the participating laboratories for one sample (no. 32/2008) used for LB proficiency testing in 2008. Absolute frequency of participants reporting positive results for each antigen are depicted by bars. Relative frequency of positive reports for the different manufacturers are shown by colored boxes within the bars. (ER, MK, VM, VT, others: anonymized abbreviations of different commercial blot manufacturers). For clinical information of samples, see Table 1.

Qualitative immunoblot test results were reported by the participants in all surveys that were performed (Tables 1 and 2). In addition, the laboratories reported the number and category of the specific IgG and IgM bands observed in their immunoblots for each of the serum samples (Figures 3(a) and 3(b)). The immunoblot results of the participating laboratories, however, showed that the individual results of the participants were not comparable in regard to the category and number of bands or the combination of bands (Figures 3(a) and 3(b)). Despite the high variability of serological test results, the microbiological interpretation of the different test constellations as reported by the laboratories was relatively homogeneous. Most participants (mean pass rate: 89.1%, range: 51.7 to 99.4%) correctly reported interpretative statements (Figure 1, Table 2) as to whether or not the assay results suggested a possible borrelial infection and whether an early or late phase of the specific antibody response was suspected.

### 3.3. False Positive and False Negative Test Results and Evaluation of Test Kit Quality

**Figure 4:**
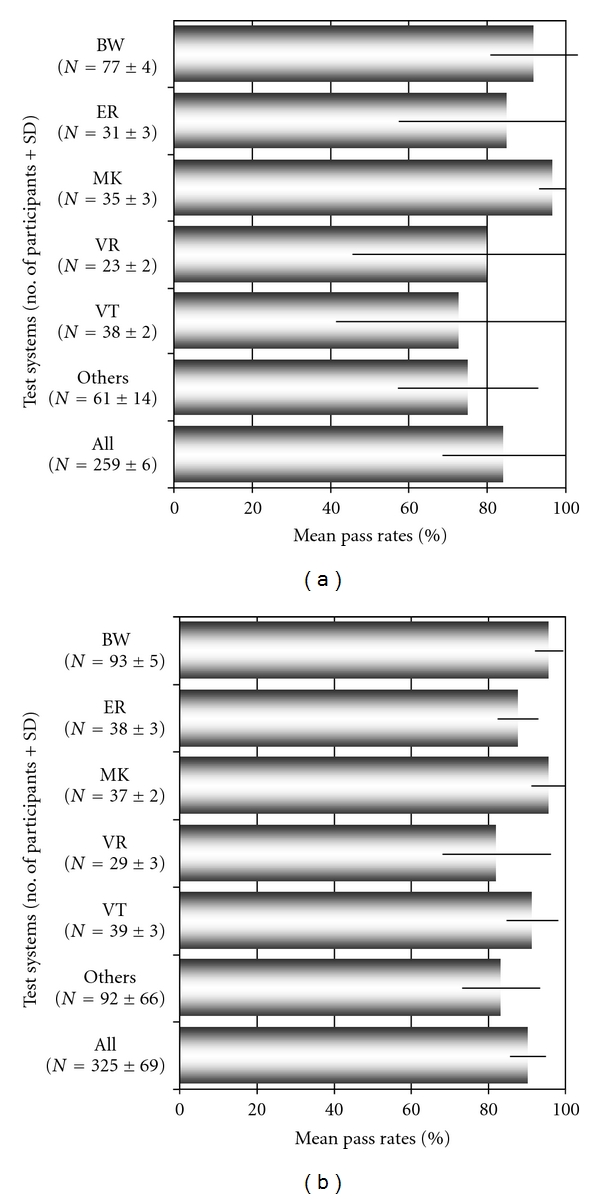
Average number of participants and average pass rates (%) with standard deviations (bars) for IgG- (a) and IgM- (b) EIAs of different manufacturers as observed between 2006 and 2008 in the German LB proficiency testing program (ER, BW, DO, MK, VR, VT, others: anonymized abbreviations of different commercial EIA manufacturers).

While using a variety of in-house tests and commercially manufactured LB test kits for the serodiagnosis of LB, participants reported a high number of false positive and false negative test results during the individual surveys. For IHA, false negative results were found in up to 33.3 of the reports and false positive results were reported in up to 30.8% of the participants. For the IgM-IFA, the rate of false negative results reached up to 35.7% and the rate of false positive results varied from 0 to 14.3% during our surveys. For the IgG-IFA, both false negative and false positive results were reported in up to 20.7 respective 58.3% of the participants. For polyvalent ELISA, the rate of false negative and false positive results ranged from 3.7 to 18% and from 0.4 to 88.2%. Class-specific ELISA testing also produced false positive results in up to 6.6% of the IgM- and in up to 46.5% of the IgG-ELISA reports. For immunoblot testing, false negative results were detected in 0–12% of the IgG- and in 4.9–15.1% of the IgM-assay reports. False positive blot results were reported in 2.1 to 22.3% of the IgG tests and in 1.7 to 9.8% of the IgM immunoblots. As depicted in Table 2 and Figures 1 and 4, the overall performance of assays was in part highly variable during the surveys and more or less depended on the assay type and manufacturer of the diagnostic test as shown for EIA in Figures 2, 4(a) and 4(b).

### 3.4. Outpatient Data Analysis of Patients with a Coded Diagnosis of LB

**Figure 5:**
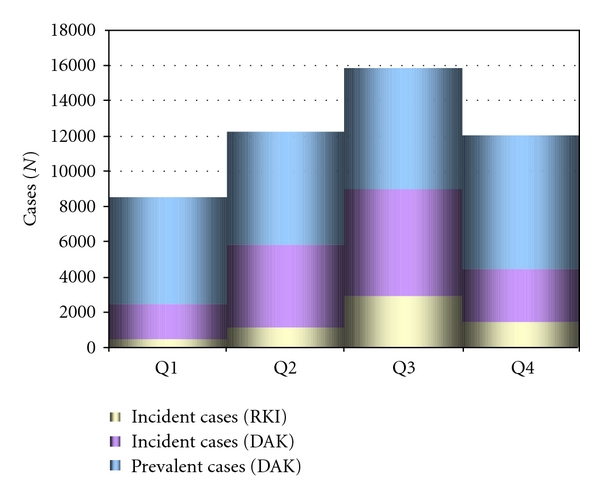
Average epidemiological annual distribution of coded incident and prevalent diagnoses of LB (incident DAK, prevalent DAK) as observed in the retrospective claims' data analysis depicted together with the average annual distribution of cases as reported by mandatory reporting (incident RKI) in the six new German states for 2007 to 2008. Q1–Q4: quarter of year.

Throughout the years 2007 and 2008, an average of 6,042,531 individuals (male: female ratio 1 : 1.6, average age: 54.7 years, male subgroup: 53.2 years, female subgroup: 55.5 years) were insured by the German health insurance provider “*Deutsche Angestellten-Krankenkasse*” (DAK). In the years 2007 and 2008, a total of 22,282 and 25,184 DAK patients were diagnosed with LB, respectively, which results in a prevalence of diagnoses of 365/100,000 (0.37%) and 442/100,000 (0.44%), respectively (average/year: 404/100,000). Sixty-six percent of diagnosed patients were female, and 34% were male. The average age of the cohort was 52.4 years (range: 0–98 years). However, a new diagnosis of LB was coded only in a fraction of these patients. For our current analysis, an incident diagnosis of LB was accepted only for the investigated individuals if a past diagnosis of LB could be excluded for both 2006 and in the preceding months of the analysed year 2007 and/or 2008. After exclusion of these (prevalent) diagnoses, 14,799 and 16,684 incident diagnoses remained for the years 2007 and 2008, respectively, leading to an average annual incidence for the diagnosis “LB” of 261/100,000 (diagnosis incidence of 242/100,000 and 279/100,000 individuals/year). Of these patients, 20,503 were female and 10,969 were male (m/f ratio: 1 : 1.9). Average age was 50.2 years (range: 1–98 years) for males and 53.6 years (range: 0–96 years) for females.  Figure 5 shows the distribution of incident and prevalent diagnoses throughout the two years analysed for this study, plotted against the data resulting from the mandatory reporting of incident LB cases for 2007 and 2008 for the eastern German states where mandatory reporting of LB is in place [9]. As depicted in Figure 5, the annual distribution of ambulatory LB diagnosis fits well into the epidemiological pattern known for incident cases from the data reported for the eastern German states [9]. Per annum, 9,303 of the newly coded patients were tested for LB serology leading to an average of 20,986 EIA tests and 12,558 immunoblots per year. Using diagnostic claims code data for these procedures, the testing resulted in a total amount of 462,980€ in diagnostic cost annually. Moreover, 19,683 antibiotic treatment courses were administered in these patients resulting in average costs of 563,508€ for antibiotic treatments per year.

### 3.5. Analysis of Sickness Fund Data on Laboratory Diagnostics in All Insured Individuals

In 2007, a total of 164,634 EIAs were ordered in 94,699 individuals. 27,362 individuals were tested further using 46,627 confirmatory immunoblots. Using diagnostic claims code data, the overall cost for serological LB diagnostics resulted in 1,267,681.8€ (7.7€/EIA) for EIA and 1,119,048€ (24€/test) for confirmatory immunoblots. In 2008, a total of 185,007 EIAs and 57,934 immunoblots were performed in 112,150 and 35,002 individuals, respectively. Overall diagnostic cost in 2008 for serological diagnostics resulted in 1,424,554€ for EIAs and 1,390,416€ for western blotting. For the same time period, only 15 cultures of *B. burgdorferi* were claimed. In both years, the highest number of tests was performed in the 2nd and 3rd quarter of the year corresponding to the highest number of incident diagnoses (Figure 5). Assuming that our insurance sample (7.4% of the German population) is representative for the whole German population of approximately 82 million [23], in the years 2007/2008 an average of 213,913 incident cases could have been expected, but 2,362,439 EIAs and 706,493 western blots would have been performed, leading to a cost of 35,146,617€ for diagnostic testing. Adjusting these cost for an additional add on of 6,106,627.94€ for the relatively higher reimbursement of laboratory cost (EIA: 23.46€, blot: 53.62€) for the known 10.5% of individuals with private health insurance the calculated average annual cost would be even higher (41,253,240.24€). Moreover, 283,912 treatments would have been administered resulting in 9,961,865€ for antibiotic therapies. When extrapolating the findings from our cohort to the German population as a whole, this rather conservative calculation would translate into testing of 1,397,628 individuals for suspected LB and total annual costs of 51,215,105.24€ for diagnostics and treatment. In contrast, focusing on incident LB cases, an average of 213,913 individuals would have been tested annually with 285,165 EIAs and 170,646 western blots leading to a cost of 6,291,290€ for diagnostic testing. Adjusting these cost for an additional add on of 1,002,617.17€ for the relatively higher reimbursement of laboratory cost (EIA: 23.46€, blot: 53.62€) for the known 10.5% of individuals with private health insurance would lead to average annual cost of 7,293,891.67€. Moreover, 237,000 treatments would have been administered resulting in another 7,614,973€ for antibiotic treatment. This calculation would translate to necessary annual costs of only 14,908,864.67€ for the diagnostics and treatment of incident LB patients in Germany, demonstrating a potentially significant gap between cost for indicated diagnostics and the high costs resulting from less selective healthcare services provided to the population as a whole.

### 3.6. Modelling the Influence of Diagnostic Test Quality and Cost on a Population-Based Scale

Applying available seroprevalence data from Germany [24–27] and our meta-analysed proficiency testing data to estimate the impact of the quality of diagnostic testing in Germany, we used mean pass rates for EIA and immunoblot as surrogate markers for average assay sensitivity and specificity. Given a projected number of 2,362,439 EIAs annually, we assumed these tests to be half IgG- and half IgM-specific tests. Assuming an average seroprevalence of 15% for Germany [24–27] and a mean IgG-EIA pass rate of 88.6% (SD = 16.1%) for negative and of 95.4% (SD = 5.5%) for positive samples (see above), IgG screening in Germany would lead to approximately 114,460 false positive and 8,150 false negative test results. Using mean pass rates of 95.0% (SD = 4.4) for negative and 90.8% (SD = 1.9) for positive samples for the performance of IgM-EIA, IgM screening would have resulted in 50,202 false positive and 16,301 false negative test results annually. Putting into use mean pass rates for IgG and IgM immunoblot of 90.7% (SD = 7.2) (IgG immunoblot) and 95.6% (SD = 4.5) (IgM immunoblot) for negative and of 94.7% (SD = 4.6) (IgG immunoblot) and 89.1% (SD = 2.7) (IgM immunoblot) for positive samples, respectively, a given two-tier testing protocol would still result in 12,854 false positive and 26,495 false negative tests per annum on a population-based scale. In a different approach on a more test-specific basis, we also used our real world proficiency testing data (see above) to perform a fictive model calculation for a given two-tier testing protocol for specific IgG and IgM testing including computation of net sensitivities and net specificities. In the first model calculation, we used the mean proficiency testing pass rates for IgG- and IgM-ELISAs and immunoblots and obtained a net sensitivity of 90% for IgG and of 81% for IgM testing. The net specificity calculated for 98.9% for IgG and 99.8% for IgM.

In the second model calculation, we reduced the mean pass rates for IgG and IgM testing by the overall SDs as obtained throughout our surveys to adjust for slightly worse performing tests. This resulted in a loss of net sensitivity of 4.2% for IgM and of 9% for IgG and a loss of net specificity of 0.6% for IgM and of 3.4% for IgG. In this model, the reduction of net specificity and sensitivity led to an additional 192,716 immunoblot tests required and 4,625,183€ in additional cost. Moreover, the relatively small difference in net specificity added up to 6,191 additional false positive test results for IgM and 34,913 false positive test results for IgG. Finally, during our proficiency testing trials, we obtained a mean pass rate of 83.9% (SD = 17.2) in positive and 95.8% (SD = 4.5) in negative samples for the correct diagnostic interpretation of laboratory test results. Given these pass rates, the serological testing of 1,397,628 individuals annually for LB as projected above would lead to a misinterpretation rate of up to 12% (*N* = 167,715) even when it comes to the simple diagnostic question whether a positive or negative LB serology is present or whether an early or late phase of the antiborrelial immune response can be found.

## 4. Discussion

Similar to the situation in the United States with 20,000–30,000 LB cases reported annually [28], LB remains an important and very common indigenous infectious disease in Germany and Europe [8]. Although high morbidity can be expected from the disease due to the large number of cases and the potentially protracted course of the infection, little effort has been invested so far in investigations elucidating the epidemiological and financial impact of LB on the German health care system. This lack of health service data is striking when comparing LB with other common infectious diseases such as community-acquired pneumonia or nosocomial infections and is probably due to the readily available antimicrobial treatment options and the lack of mortality in LB. Likewise, such investigations are missing for most other parts of Europe making it difficult to assess the true dimension of the underlying medicoeconomical burden. Here, we used retrospective data analysis to examine (i) the epidemiology of LB, (ii) the quality of diagnostic testing, and (iii) the cost for diagnostic testing in Germany.

With official numbers absent for most parts of Germany, established mandatory reporting for 2007 and 2008 revealed about 5,624 annual incident cases (Figure 5) of LB (mainly erythema migrans) in the six new federal states (population: 16,507,263) in the north-eastern part of Germany [9]. Extrapolating these findings to the German population of 82 million, this would translate into approximately 27,958 incident cases for the entire country annually. This strongly suggests significant underreporting even when compared to the commonly cited numbers of 60,000–100,000 new LB cases per year as calculated from information available from previous seroprevalence investigations [8]. Thus, in a different approach, here, we used a retrospective outpatient data analysis performed between 2007 and 2008 on a cohort of 6,042,351 individuals insured by a German health care provider (DAK) to estimate the incidence and prevalence of LB in Germany by identifying the number of incident and prevalent diagnoses as a surrogate.

In the first step of our investigation, using these data, we were able to analyze a very large patient sample spread over the entire country and including all age groups. Importantly, possible bias due to recall, nonresponse, or the diagnostic process of attention is markedly reduced in such datasets compared to other study types [29]. Following strict definitions for the identification of “incident” and “prevalent” diagnoses, we identified 23,733 patients with a coded diagnosis of LB per year (prevalence: 404/100,000). An incident diagnosis of LB was coded in 14,799 and 16,684 individuals for the years 2007 and 2008, respectively, resulting in an incidence of 261/100,000 cases annually in the DAK cohort. Although the extrapolation of these numbers may lead to an overestimation due to clinical misdiagnosis and/or miscoding, our findings translate into 213,912 annual incident cases on a population-wide scale, which suggests more LB cases in Germany than projected previously in the available literature dealing with this topic.

Several recently performed interlaboratory studies have compared the diagnostic performance of serological tests for LB [5, 11, 30–35]. Such investigations, however, can provide only limited information on the overall performance and relative accuracy of diagnostic testing in general on a nationwide scale. Therefore, in a second step our study was aimed at supplying additional data on the quality of LB diagnostics at the national level over a well-defined period of time. In addition, we tried to identify limitations and overuse of current diagnostic approaches to LB over a well-defined period of time paralleling our retrospective patient data analysis as outlined above. According to most guidelines, LB serology should only be performed to support clinical diagnosis, not as a primary basis for making diagnostic and/or treatment decisions [4]. As shown in this investigation and previous studies, serologic testing can be flawed by problems with both sensitivity and specificity [11]. Not unexpectedly, different methodological approaches in themselves can result, to some extent, in substantial differences with regard to test quality. Currently, a large variety of serological tests for the detection of LB are available in the European market, supplied by an increasing number of manufacturers. In the United States, a complex regulatory system for new in vitro diagnostics is in place which requires the manufacturer to compare its product substantially against an established device that has already been cleared by the FDA [36, 37]. In Europe, the institution of the new European IVD directive in 2000 did not legally insist in extensive, independent, and continuous *clinical* evaluation of commercially available serological test kits for LB before placing in vitro diagnostic tests on the market [38]. Instead, quality standards for the production quality and safety are enforced for in vitro diagnostic tests in their intended use [38, 39], and; consequently, test remakes are increasingly pushed onto the market. This trend is also supported by our observation showing a steady increase (~50%) of new test formats such as line blots and CLIA during the study period in the years 2006–2008. Currently, at least 55 different companies provide diagnostic tests for LB in Germany alone. Therefore, routine evaluations of microbiological laboratories by external quality control measures for LB serology appear to be attractive datasets to learn more about the relative frequency of certain test applications (i.e., EIA and immunoblot) in the diagnostic market, the amount of test standardisation, and the quality of performance in diagnostic infection serology [11, 19]. From 2006 to 2009, in Germany, between 360 and 392 microbiological laboratories took part in our proficiency testing surveys. Similar to findings of other investigations, most laboratories still relied on two-tier testing with EIA and immunoblot throughout the study period. Although qualitative testing by EIA and immunoblot showed mean pass rate ranges from 82.2 to 89.2% (Figure 1), quantitative EIA results and analysis of immunoblot banding patterns, however, demonstrated a very low degree of interassay standardisation (Figures 2 and 3). In addition, as already described in prior studies [11, 34, 35], a high number of both false negative and false positive test results became obvious from our surveys and was in part correlated with the diagnostic method, the manufacturer (Table 2, Figures 1 and 4), and the amount of specific antibodies present in different sera (Table 1). Our findings confirm the assumption that, in the routine laboratory, the quantity of detected antibody measured in titers or quantitative EIA results and, similarly, the number and category of specific immunoblot bands can vary greatly for the same sample. In addition, changes in qualitative and quantitative serologic test results may be misleading and can emerge simply by using different assay systems in different laboratories. As a consequence of the findings in this investigation and other recent studies, a more general implementation of diagnostic criteria for the interpretation of immunoblot results as suggested by expert recommendations [13] seems increasingly difficult in light of the relatively high assay variability (Figures 2 and 3). Most importantly, correlating the activity of LB and the success of subsequent therapies with quantitative serological testing as well as with qualitative changes in the test results as attempted by some physicians appears clearly unreliable. The extreme variability of test results reported by the participants in our surveys is concordant with the few available international studies on this topic [11, 35, 40–42]. To improve the value of LB serology in the routine microbiological laboratory, promotion of better interassay standardisation of the commercially available test kits is necessary [13, 35, 42] by implementing standards and procedures as suggested earlier by the Centers for Disease Control and Prevention (CDC) and the Association of State and Territorial Public Health Laboratory Directors (ASTPHLD) conference on the serological diagnosis of LB [43]. Most importantly, more detailed and independent clinical evaluation of assays should be legally required before placing such products on the market [19].

So far, very few studies have examined the economic impact of quality and frequency of LB diagnostic testing on health care on the national level in the US and Europe. One study examined the medical and economical burden of LB in the United States. Using a decision analysis model and an estimated incidence of 4.7 LB cases per 100,000 persons led to direct and indirect costs including diagnostic testing of about 2.5 billion US Dollars over 5 years for the US [44]. For the state of Maryland, Zhang et al. calculated direct medical costs including diagnostics of 2,970 US Dollars plus indirect costs of 5,202 US Dollars per LB patient [45]. This would add up to approx. 200 million US Dollars per year for the United States. Another study estimated the cost of LB for the Scottish health care system. Although this study was limited to laboratory testing only, the authors estimated a total of GBP 47,000–615,000 for Scotland which seems to be a high financial impact for a country with a relatively low LB incidence [46]. All mentioned investigators used variable assumptions and economical models to assess the general cost of LB making it difficult to directly compare their findings to the results of our study. The results of these studies, therefore, cannot simply be transferred to the German health system due to differences in the epidemiology of the disease, of the methodological approaches, and in the health systems. Besides the cost for physician visits, consultation, and therapy, clearly, the cost for diagnostic testing, represents one of the major variables when calculating direct medical costs of LB on a population-wide scale. This is why, in the third step of our investigation, we tried to estimate the quantity and cost of diagnostic testing in Germany by modelling the combined information obtained from the DAK dataset and from the results of the regular German LB proficiency testing surveys run by INSTAND biannually to estimate the cost and medical quality of laboratory diagnostics on a nationwide level. By assessing the diagnostic frequency, quality, and cost of LB diagnostics in Germany, we estimated that 2,371,887 EIA tests and 709,331 western blots are performed annually. When modelling the influence of test quality for a given two-tier testing protocol including the calculation of net sensitivities and net specificities, it became obvious that using our real world proficiency testing data such tests would result in 12,854 false positive and 26,495 false negative test results annually on a population-wide scale. In this model, a small reduction in net specificity led to an additional 192,716 immunoblot tests required and an amount of 4,625,183€ in extra cost. Moreover, the small reduction in net specificity added up to 6,191 additional false positive test results for IgM and 34,913 false positive test results for IgG. Finally, given the average pass rates for the correct diagnostic interpretation of laboratory test results obtained during our proficiency testing trials, the serological testing of 1,397,628 individuals annually as projected above would lead to a misinterpretation rate of up to 12%.

By further extrapolating the findings from our cohort of 6.04 million individuals insured by a statutory health insurance provider and adjusting our findings for 10,5% of privately insured patients with higher reimbursement, we project an overall expected cost of 51,215,105.24€ for LB serologic testing and treatment in Germany. These figures do not include patients' expenditure for nonrecommended tests (e.g., lymphocyte-transformation tests, LTT) which are not reimbursed but have to be paid out of the pocket. Similarly, in a study on LB management in primary care practices in Maryland USA, both diagnostic and therapeutic efforts were heavily overused [47]. In addition, Ramsey et al. showed that 80% of serology tests for Lyme borreliosis were regarded as inappropriate in a retrospective analysis in Wisconsin, USA [48]. Our findings on cost also come close to the study of Tugwell et al. who estimated 2.8 million tests in the US/year, leading to 100 million USD/year for serological testing [49]. The somewhat higher cost for testing in the US can be explained due to higher expenditure per test compared to the German situation. However, with our projected incidence of 214,000 cases per year, the overall frequency and cost of diagnostic testing and treatment clearly suggests a high amount of potentially inappropriate healthcare services in patients with a suspected or confirmed diagnosis of LB.

## 5. Conclusion

Our study is the first investigation of its kind in Germany and looks into the medical and economical burden of LB testing for the German healthcare system. Although suggesting a high amount of inappropriate diagnostic healthcare services, our analysis also shows limitations as it is focused on retrospective investigations of proficiency testing surveys and secondary claims data including external quality control datasets, coded diagnoses, and diagnostic and therapeutic services relevant for physician claims. Given our secondary data study design, other potential biases which cannot be accounted for including missing information on services provided outside the statutory health insurance and absence of information related to care provided which does not lead to (additional) claim codes (i.e., multiple visits within a quarter) [29, 50]. However, the findings coming from the GILEAD project are a first approximation of health care services provided related to LB. They will help to assess and better tailor the quality standards for diagnostic tests and the economics of current and future disease management and prevention programs for LB. Given the ongoing problems in Germany with the clinical management of LB, it seems important to closely monitor and evaluate health care utilization patterns including diagnostics and treatment for LB patients to both facilitate a better understanding of existing care and design intervention approaches to improve the clinical management for such patients.

## Figures and Tables

**Table 1:** German LB proficiency testing program: characteristics of selected serum samples as determined by the reference laboratories of the BISSGG.

Sample	PHA	EIA (poly valent)	IFA- IgG	IFA- IgM	EIA- IgG	EIA- IgM	CLIA- IgG	CLIA IgM	Line blot	Immuno-blot IgG	Immuno-blot IgM	Clinical information (time of sampling after therapy)
31/2006	N (<80)	N	N (<40)	N (<20)	N	N	N	N	N	N	N	Syphilis stage I (4 yrs)
32/2006	P (1280)	P	P (320)	B/P (20)	P	B/P	P	B/P	P	P	B/P	Recent Lyme arthritis (1 yr)
61/2006	N (<80)	N	N (<40)	N (<20)	N	N	N	N	N	N	N	Healthy blood donor
62/2006	P (320)	P	B/P (80)	P (320)	B/P	P	P	P	P	B/P	P	Morbus Bannwarth (1.5 mo)

21/2007	P (160)	B/P	B/P (80)	N/B/P (≤40)	B/P	N/B/P	B/P	N/B/P	B/P	B/P	B/P	Seropositive but asymptomatic donor, several tick bites in recent medical history
22/2007	N (<80)	N	N (<40)	N (<20)	N	N	N	N	N	N	N	Healthy blood donor
61/2007	N (<80)	N	N (<40)	N (<20)	N	N	N	N	N	N	N	Syphilis stage II (8 weeks after treatment)

62/2007	P (1280)	P	P (320)	N (<20)	P	N	P	N	P	P	N/B	Seropositive but asymptomatic donor, several tick bites in recent medical history
31/2008	N (<80)	N	N (<40)	N (<20)	N	N	N	N	N	N	N	Healthy blood donor

32/2008	P (320)	P	P (160)	P (80)	P	P	P	P	P	P	P	Morbus Bannwarth, CSF pleocytosis, specific CSF antibody index (AI): 6.3

61/2008	P (1280)	P	P (160)	N/B (≤20)	P	N	P	N	P	P	N	Lymphocytoma (2 mo)
62/2008	N (<80)	N	N (<40)	N (<20)	N	N	N	N	N	N	N	Healthy blood donor

*Legend: P, positive, B, borderline, and N, negative. For some samples, combinations (i.e., N & B, B & P, or N/B/P) were accepted. Median titers as determined by the reference laboratories are given in parentheses.

**Table 2:** Mean pass rates of proficiency testing participants in regard to samples tested and assay system used.

Sample	IHA		Polyvalent EIA	IgG- EIA	IgM- EIA	IgG- IFA		IgM- IFA		IgG Blot	IgM blot	Line blot	IgG- CLIA	IgM- CLIA	Diagnostic Comment
Positive samples	Qual.	Quant.	Qual.	Qual.	Qual.	Qual.	Quant.	Qual.	Quant.	Qual.	Qual.	Qual.	Qual.	Qual.	Qual.

32/2006	100	88.9	95.5	97.0	90.7	93.5	93.5	64.3	61.5	97.1	95.1	97	95.7	11.5	98
62/2006	66.7	76.9	82	83.3	92.0	88.0	85.7	90.9	78.9	89.2	89	83.3	100	71.4	88.7
21/2007	93.3	82.4	90.9	96.1	95.7	79.3	80.8	100	100	93.9	89.4	94.1	96.8	100	51.7
62/2007	85.7	66.7	91.4	99.2	93.7	95.8	81.8	71.4	75	100	84.9	96.9	100	91.2	97.7
32/2008	78.6	78.6	96.3	97	91.4	95.7	81	80	75	88	88.5	96.2	100	100	71.0
61/2008	100	100	96.2	99.6	81.4	90.9	77.3	89.5	78.9	100	87.7	96.7	100	91.8	96.5

**Mean**	**87.3**	**82.3**	**92.1**	**95.4**	**90.8**	**90.5**	**83.4**	**82.7**	**78.2**	**94.7**	**89.1**	**94.0**	**98.8**	**77.7**	**83.9 **

Negative samples	Qual.	Quant.	Qual.	Qual.	Qual.	Qual.	Quant.	Qual.	Quant.	Qual.	Qual.	Qual.	Qual.	Qual.	Qual.

31/2006	94.1	88.9	95.9	93.9	95.5	93.5	93.5	85.7	84.6	97.9	94.6	93.9	100	100	98.3
61/2006	100	100	99.6	100	93.4	92.0	90.5	95.5	94.7	96.1	95.6	100	100	96.4	99.4
22/2007	100	100	87.9	88.8	93.7	62.1	53.8	92.6	92	84.6	90.2	75	100	100	92.4
61/2007	69.2	71.4	11.8	53.5	94.1	41.7	40.9	95.2	95	77.7	96.9	87.9	100	91.2	87.4
31/2008	100	100	96.2	98.9	96.4	91.3	90.5	100	95	94.4	98.3	100	100	97.4	99.4
62/2008	100	100	96	96.8	97.1	86.4	81	100	94.4	93.8	98.2	91.7	100	93.9	98.1

**Mean pass rates**	**93.8**	**93.3**	**81.2**	**88.6**	**95.0**	**77.8**	**75.0**	**94.8**	**92.6**	**90.7**	**95.6**	**91.4**	**100**	**96.5**	**95.8**
